# Comparing Current and Next-Generation Humanized Mouse Models for Advancing HIV and HIV/*Mtb* Co-Infection Studies

**DOI:** 10.3390/v14091927

**Published:** 2022-08-30

**Authors:** Madeleine Lepard, Jack X. Yang, Sam Afkhami, Aisha Nazli, Anna Zganiacz, Shangguo Tang, Margaret Wa Yan Choi, Fatemah Vahedi, Alexandre Deshiere, Michel J. Tremblay, Zhou Xing, Charu Kaushic, Amy Gillgrass

**Affiliations:** 1 McMaster Immunology Research Centre, Michael G. DeGroote Institute for Infectious Disease Research, Department of Medicine, McMaster University, Hamilton, ON L8S 4K1, Canada; 2 Department of Pathology and Molecular Medicine, McMaster University, Hamilton, ON L8N 3Z5, Canada; 3 Axe des Maladies Infectieuses et Immunitaires, Centre de Recherche du CHU de Québec-Université Laval, Pavillon CHUL, Québec City, QC G1V 4G2, Canada

**Keywords:** HIV, TB, humanized mice, mouse models of infectious disease

## Abstract

In people living with HIV, *Mycobacterium tuberculosis* (*Mtb*) is the major cause of death. Due to the increased morbidity/mortality in co-infection, further research is urgently required. A limiting factor to research in HIV and HIV/*Mtb* co-infection is the lack of accessible in vivo models. Next-generation humanized mice expressing HLA transgenes report improved human immune reconstitution and functionality, which may better recapitulate human disease. This study compares well-established huNRG mice and next-generation HLA I/II-transgenic (huDRAG-A2) mice for immune reconstitution, disease course, and pathology in HIV and TB. HuDRAG-A2 mice have improved engraftment of key immune cell types involved in HIV and TB disease. Upon intravaginal HIV-1 infection, both models developed significant HIV target cell depletion in the blood and tissues. Upon intranasal *Mtb* infection, both models sustained high bacterial load within the lungs and tissue dissemination. Some huDRAG-A2 granulomas appeared more classically organized, characterized by focal central necrosis, multinucleated giant cells, and foamy macrophages surrounded by a halo of CD4+ T cells. HIV/*Mtb* co-infection in huNRG mice trended towards worsened TB pathology and showed potential for modeling co-infection. Both huNRG and huDRAG-A2 mice are viable options for investigating HIV and TB, but the huDRAG-A2 model may offer advantages.

## 1. Introduction

Animal models are effective tools for studying human infectious diseases as well as for investigating and designing appropriate interventions. However, in the context of certain human-tropic pathogens such as human immunodeficiency virus (HIV-1), the lack of human immune cells can restrict the scope of in vivo models available for research. There are currently 37.7 million people living with HIV, with 1.5 million newly acquired infections each year [1]. Of particular concern are the high rates of co-infection in individuals with *Mycobacterium tuberculosis* (*Mtb*) [2], where HIV co-infection increases the risk of progressing to active tuberculosis (TB) by over 20-fold [3]. The primary cause of death in individuals living with HIV is co-infection with *Mtb*, resulting in 214,000 deaths globally in 2020 alone [4]. Co-infection is deadly and frequent given the high geographical overlap of these pathogens, particularly in Sub-Saharan Africa [5]. The increased morbidity and mortality of co-infection, as well as the rise in drug-resistant strains of *Mtb,* has resulted in continued challenges in treatment and disease eradication, and thus, further research is urgently required [6,7,8].

While standard inbred mice cannot be used for HIV-1 research due to the lack of human CD4+ T cells required for HIV-1 infection, they have contributed greatly to the investigation of TB. Non-human primate (NHP) have been very useful for both HIV and TB research, but host-restriction factors remain in these models. Additionally, the feasibility of NHP models is limited due to ethics and high cost, which restricts their widespread use [9]. Therefore, small animal models sustaining human immune cells are critical to the advancement of HIV research and HIV/*Mtb* co-infection research. Humanized mouse (hu-mouse) models address many of the challenges of NHP models and have demonstrated their utility for infectious disease studies. After decades of development, some of the commonly used immunocompromised strains for creating humanized mice (hu-mice) include the Rag1^null^Il2rg^null^ or Rag2^null^Il2rg^null^ (BRG, also known as DKO), NOD.Cg-Prkdc^scid^Il2rg^tm1Sug^ (NOG), NOD.Cg-Prkdc^scid^Il2rg^tm1Wjl^ (NSG), and NOD.Cg-Rag1^tm1MoM^Il2rg^tm1Wjl^ (NRG) mice engrafted with hematopoietic stem cells (HSCs). These are the current generation of hu-mice and have demonstrated human CD45+ immune cell reconstitution, including human CD4+ and CD8+ T cells, B cells, and low to absent myeloid populations. These models also have certain limits, such as low levels of successful reconstitution (50% of mice successfully engrafted), relatively low levels of total human immune cells and little-to-no isotype switched antibodies, reflecting limits to functionality [10,11,12]. To better recapitulate certain aspects of human immune responses, next-generation hu-mice expressing various transgenic modifications have been developed and are reviewed extensively elsewhere [13,14]. When engrafted with HLA-matched HSCs, several HLA-transgenic hu-mouse models, such as the humanized DRAGA (HLA-DRB1*04:01/*02:01) [15,16], NSG-A2 [17], BRGS-A2-DR2 [18], NOG-DR4 [19], and NOK-B51Tg [20], have demonstrated better lymphoid development and improved immune functionality (isotype switched antibodies and T cell functionality) either upon stimulation or pathogen challenge when compared to their non-transgenic counterparts. Overall, these improvements in HLA-transgenic mice offer researchers an in vivo model for infectious diseases that may better recapitulate human immune responses and can be available for widespread use.

Many hu-mouse models have become a cornerstone of HIV-1 research and recapitulate many aspects of human HIV infection, including mucosal infection, characteristic drop in CD4+ T cells and response to human therapeutics such as antiretroviral therapy [13,21,22,23,24,25]. Although TB has been successfully studied and modeled in several standard mouse models [26,27,28], hu-mouse models of TB have revealed advantages such as the formation of more human-like granulomas, displaying features such as organized immune cells surrounding a caseating and necrotic core [29,30,31,32]. Additionally, a recent study using huNSG-bone marrow, liver, thymus (BLT) mice (engrafted with matching fetal tissues and HSCs) demonstrated great potential for hu-mice to be used for *Mtb* infection and HIV/*Mtb* co-infection studies [29,33]. Many aspects of the human TB and HIV disease pathology were observed [29,33]. These studies demonstrated potential for other hu-mouse models, such as next-generation HLA-transgenic strains engrafted with the more feasible HSC-method of engraftment, to be used as a pre-clinical exploratory model to gain insight into co-infection pathologies.

Here, we compared the current generation HSC-method (huNRG) and a next-generation HSC-method HLA transgenic model (huDRAG-A2) to determine their utility and applicability in the study of HIV and TB. HuDRAG-A2 mice are HLA transgenic for HLA I A2.1 and HLA II DR4 and have been reconstituted with HLA-matched stem cells. We found that huDRAG-A2 mice have an increased frequency of certain immune cells (hCD4, hCD14) in comparison to huNRG mice. Both huNRG and huDRAG-A2 mice are infected by HIV and show similar viral loads and a characteristic drop in human CD4+ T cells in both the blood and in the mucosal tissues. We show for the first time infection of huDRAG-A2 mice with H_37_Rv *Mtb*. Both huNRG and huDRAG-A2 have similar *Mtb* load in the lung and dissemination to the spleen. Overall, granuloma formation in huNRG and huDRAG-A2 have characteristics of human granulomas, with the huDRAG-A2 mice having granulomas with a focal necrotic core containing human macrophages and an immune cell ring surrounding it. Our results show that both models recapitulate multiple aspects of human HIV and TB infection but that the huDRAG-A2 may offer advantages in terms of higher immune cells (CD4 T cells, macrophages) as well as granulomas with a more classic human pathology, which may be beneficial in the future of co-infection and in studies that depend on the immune response. In a pilot HIV/*Mtb* co-infection in huNRG mice, we observed a trend of increased pathology with co-infection, thus displaying the promise of these models for future studies. Creating improved models can help to better model disease susceptibility, disease course, histopathology and immune responses to enable the development of therapeutics and vaccines.

## 2. Materials and Methods

### 2.1. Generation of Humanized Mice

NOD-Rag1^null^ IL2rg^null^ (NRG) mice (stock ID# 007799), as well as NRG mice with transgenes for human HLA-DR4/DRB1*04:01 (DRAG) (stock ID# 007799) were obtained from Jackson laboratories (Bar Harbor, ME, USA). Animal procedures were all approved by the Animal Research Ethics Board and followed CCAC guidelines (AUP# 21-12-39, 21-04-12, 18-06-26 and 17-06-29). NRG mice with transgenes for human HLA-A2.1/A*02:01 (A2) were obtained as a generous gift from Dr. Ali Ashkar (McMaster University, Hamilton, ON, Canada) (see Supplementary Methods). DRAG homozygotes were bred with A2 homozygous NRG mice to create mice with both the HLA-DR4 and HLA-A2 transgenes (DRAG-A2). Genotyping of mice was performed by polymerase chain reaction (PCR) on DNA extracted from ear tissue using the DNeasy Blood and Tissue Kit (QIAGEN, Toronto, ON, Canada) to confirm the expression of both the DRAG and A2 alleles (see Supplementary Methods).

Both NRG and DRAG-A2 mice were engrafted with CD34+ hematopoietic stem cells (HSC) isolated from human umbilical cord blood (HiREB approved ethics #13-813-T). The cord blood samples were depleted of red blood cells with HetaSep (StemCell, Vancouver, BC, Canada). CD34+ HSCs were enriched from the remaining nucleated cells using the RosetteSep Human Hematopoietic Progenitor Cell Enrichment Cocktail CD34 negative selection kit (StemCell, Vancouver, BC, Canada). CD34+ HSCs were then separated using Lymphoprep density gradient centrifugation (StemCell, Vancouver, BC, Canada) and frozen for storage in liquid nitrogen in CryoStor CS10 freezing media (StemCell, Vancouver, BC, Canada). Cord blood samples were screened for the HLA-DR4/DRB1*04:01 and HLA-A2.1/A*02:01 alleles using MicroSSP HLA Allele Specific typing kits (OneLambda, Canoga Park, CA, USA). Results were analyzed using HLA Fusion Software (version 4.2.0) (OneLambda, Canoga Park, CA, USA) to identify possible allele pairs, then cross-referenced with the Allele Frequencies Database (http://www.allelefrequencies.net/hla.asp, last accessed on 2 March 2021) to confirm the HLA isotype. Cord blood samples identified positive for both DRB1*04:01 and A*02:01 allele isotypes were used to humanize DRAG-A2 mice (see Supplementary Methods). Newborn (24–72 h old) or adult (6–10 weeks old) NRG and DRAG-A2 mice were irradiated twice with 3 cGy 3 hours apart (pups) or once with 550 cGy (adults). 1 × 10^5^–1 × 10^6^ CD34+ HSCs was then injected either intrahepatically in 30 μL phosphate-buffered saline (PBS) (pups) or intravenously in 200 μL PBS (adults) [34].

At 12-, 16- and 20 weeks post-engraftment, 50 μL of blood was collected into EDTA-coated blood collection tubes (BD Biosciences, Toronto, ON, Canada) to quantify human immune cell reconstitution using flow cytometry. Erythrocytes were lysed using an ACK lysis buffer (Quality Biological, Gaithersburg, MD, USA), and the remaining cells were treated with both anti-human Fc Receptor Binding Inhibitor and anti-mouse CD16/CD32 antibodies (eBiosciences, San Diego, CA, USA). The cells were then stained with an antibody cocktail (mCD45-AlexaFluor 700, hCD45-Pacific Blue, hCD3e-Qdot 605, hCD4-PerCP-Cy5.5, hCD8a-PE-Cy7, hCD19-PE, hCD14-AlexaFluor 647, eBiosciences, San Diego, CA, USA; hCCR5-FITC, BD Biosciences, Toronto, ON, Canada), followed by fixable viability dye (APC-eFluor 780, eBiosciences, San Diego, CA, USA) (Table S1) and then the samples were run on the Cytoflex LX flow cytometer (Becton Dickson, Franklin Lakes, NJ, USA) equipped with a flow rate calibrator and analyzed using FlowJo software (version 10.7.1) (Becton Dickson, Franklin Lakes, NJ, USA). Mice with at least 10% or 50,000 per mL hCD45+ leukocytes in the blood were selected for subsequent experiments (Tables S2–S4) [21]. 

### 2.2. Intravaginal HIV Infection of Humanized Mice

An R5-trophic strain of HIV-1, NL4.3-Bal-Env, was generated by transient transfection of 293T human embryonic kidney cells (HEK 293T), as previously described [21]. Briefly, HEK293T cells were plated and transfected after 24 h with pNL4.3 Bal Env HIV-1 vector (50 ug plasmid per 4 × 10^6^ cells). After 48 h of transfection, viral supernatants were collected, ultracentrifuged (28,000G for 90 min at 4 °C) and reconstituted in PBS [21]. One week prior to infection, successfully engrafted (%hCD45+ of total leukocytes > 10%) female huNRG (N = 5) and huDRAG-A2 (N = 3) mice (Table S3) were treated with 2 mg subcutaneous depot-medroxyprogesterone acetate (DMPA) to induce diestrus, which was confirmed 5 days post-DMPA by microscopy of vaginal lavage [35,36,37]. Mice were anesthetized with 100 mg/kg ketamine and 10 mg/kg xylazine via intraperitoneal (IP) injection, and the vaginal canal was swabbed before intravaginal administration of 3.6 × 10^5^ TCID50 of NL4.3-Bal-Env HIV-1. Vaginal lavage and blood plasma were collected at 2, 4, 6 and 8 weeks post-infection for viral load quantification using RT-qPCR. Viral RNA was extracted from 50 μL of plasma or vaginal wash using the QIAamp MinElute Virus Spin Kit (QIAGEN, Toronto, ON, Canada) and RT-qPCR was performed using the SensiFAST Probe Hi-ROX One-Step Kit (Bioline, Taunton, MA, USA) using primers specific for the HIV long terminal repeat (LTR) promoter (forward primer: 5′-GCCTCAATAAAGCTTGCCTTGA-3′; Reverse primer 5′-GGCGCCACTGCTAGAGATTTT-3′; reporter probe: 5′-AAGTAGTGTGTGCCCGTCTGTTRTKTGACT-3′, 5′ reporter 6-FAM and 3′ quencher TAMRA). The threshold of detection of the assay was 1500 HIV-1 RNA copies/mL. Whole blood was also collected at 8 weeks post-infection for flow cytometric analysis, as previously described. Cells were stained with hCD45-PacBlue, mCD45-Alexa Fluor 700, hCD3e-Qdot 605 and hCD4-PerCP-Cy5.5 antibodies (eBiosciences, San Diego, CA, USA).

### 2.3. Immunohistochemistry

The lung and vaginal tissues were collected and fixed in 10% formalin. Human CD4+ T cell and CD68+ macrophage immunohistochemistry staining was performed. Briefly, sections were deparaffinized and boiled in Tris/EDTA (pH = 9) for 20 min in a microwave. After blocking with 5% bovine serum albumin (BSA), sections were incubated with 1:50 mouse anti-human CD4 monoclonal antibodies (clone 4B12) (Leica Biosystems, Nussloch, Germany) or mouse anti-human CD68 monoclonal antibodies (clone PG-M1) (Dako, Glostrup, Denmark) for 1 h at room temperature, followed by visualization using the Bond Polymer Refine Red Detection kit (Leica Biosystems, Nussloch, Germany) on an automated Leica Bond RX autostainer (Leica Biosystems, Nussloch, Germany). Human tonsil and non-humanized NRG mouse lung sections were used as positive and negative controls, respectively (Figure S1). To quantify immune cell depletion in the lung and vagina, an image was taken of each slide using the ZEISS Axiolmager M2 microscope (Zeiss, Toronto, ON, Canada) and the ZEN Pro software (version 2.3) (Zeiss, Toronto, ON, Canada) at 5× magnification. Next, five additional images were taken at five randomly picked areas at 20× magnification. With the “Cell Counter” tool on the Fiji ImageJ software (version 2.3.0/1.53q), the CD4+ and CD68+ cells were counted manually on the five areas of each tissue section. The mean cell count of the five areas was used to represent the CD4+ or CD68+ cell count in the tissue section of each mouse. For the spleen, an image was taken using the ZEISS Axiolmager M2 microscope (Zeiss, Toronto, ON, Canada) and the ZEN Pro software (version 2.3) (Zeiss, Toronto, ON, Canada) at 5× magnification. Images were then processed using Fiji ImageJ software (version 2.3.0/1.53q) (Bethesda, MD, USA) using the mean grey intensity method as described in [38]. First, background staining was removed via color deconvolution (to implement stain separation) and then thresholding was performed (to divide the images into foreground and background pixels). Following color deconvolution and thresholding, the mean grey intensity was measured to quantify CD4 immunohistochemical staining as described in [38].

### 2.4. Intranasal TB Infection of Humanized Mice

Well-engrafted (%hCD45 of total leukocytes > 10%) male and female huNRG and huDRAG-A2 mice were infected intranasally with *Mtb* (Table S4). Mice were anesthetized briefly with gaseous isoflurane and infected intranasally with a target dose of 1 × 10^4^ (confirmed dose by colony forming assay of 5 × 10^3^–2 × 10^4^ CFU *Mtb* H_37_Rv) (N = 6 huNRG and 3 huDRAG-A2). At 4 weeks post-infection, the lung and spleen were collected for pathological scoring and bacterial load quantification using CFU enumeration. Briefly, lung and spleen were homogenized mechanically and plated in 10-fold serial dilutions on Middlebrook 7H10 agar plates containing Middlebrook oleic acid-albumin-dextrose-catalase (OADC) enrichment and supplemented with 5 μg/mL ampicillin and 50 μg/mL cycloheximide (Sigma-Aldrich, Oakville, ON, Canada). Plates were incubated for 3 weeks at 37 °C, and the colonies were counted to quantify the bacterial load. A small portion of the lung and spleen was also analyzed using flow cytometry, as previously described [39]. Briefly, cells were extracted by digestion in a type 1 collagenase solution for 1 h (10 U/mL)(Gibco, Waltham, MA, USA), then tissues were passed through a 100 μm cell strainer (VWR, Mississauga, ON, Canada) and then stained with an antibody cocktail to measure macrophage populations (mCD45-AlexaFluor 700, hCD45-Pacific Blue, hCD3e-Qdot 605, eBiosciences, San Diego, CA, USA; and hCD14-BV 785, hCD11b-PerCP-Cy5.5, hCD16-FITC, hCD169-PE and hCD206-APC, BioLegend, San Diego, CA, USA) (Table S1). Splenocytes were mechanically extracted by crushing through a 100μm filter and lysed with ACK lysing buffer (Quality Biological, Gaithersburg, MD, USA) before staining with an antibody cocktail to measure T cell frequency (mCD45-AlexaFluor 700, hCD45-Pacific Blue, hCD3e-Qdot 605, hCD4-PerCP-Cy5.5, hCD8-PE-Cy7, hCD19-PE; eBiosciences, San Diego, CA, USA) (Table S1) and analyzed using FlowJo software (version 10.7.1) (Becton Dickson, Franklin Lakes, NJ, USA).

### 2.5. Analysis and Quantification of Histopathology of TB-Infected Humanized Mice

Lung and spleen tissue were collected and fixed in 10% formalin before hematoxylin and eosin (H&E), acid-fast bacilli (AFB) staining, as well as human CD4+ T cell and CD68+ macrophage immunohistochemistry, as previously described. Histology sections were analyzed by a pathologist. H&E stained lung samples from *Mtb*-infected huNRG and huDRAG-A2 mice were analyzed to quantify granulomatous tissue. The slides were viewed using Aperio ImageScope (version 12.4.6) (Leica Biosystems, Nussloch, Germany) and snapshot images were taken of each section. These images were then examined on Fiji ImageJ software (version 2.3.0/1.53q) (Bethesda, MD, USA). With the “freehand selection tool”, the entire lung tissue, as well as the granulomas, were traced, and their areas were measured in mm or μm using the scale bar output setting. The mean percentage of granulomatous tissue of the total lung tissue was calculated for each sample. 

### 2.6. HIV/Mtb Co-Infection of huNRG Mice

To model the single HIV-1 infection studies, all female huNRG mice (*Mtb*-only group, N = 2; co-infected group, N = 3) were given 2 mg DMPA subcutaneously, and diestrus was confirmed microscopically, as described above. One week after the DMPA treatment, huNRG mice in the HIV/*Mtb* co-infected group (N = 3) received 3.6 × 10^5^ TCID50 NL4.3-Bal-Env HIV-1 in a 25 µL volume intravaginally, as described above. Plasma viral load was assessed at 2 and 3 weeks post-HIV infection to confirm successful HIV infection. 3.5 weeks post-HIV infection, all huNRG mice were infected intranasally with a target low dose of 1 × 10^3^ (actual dose 2.3 × 10^3^) CFU of H_37_Rv *Mtb* in a 25 µL volume. Tissues were collected 4.5 weeks post-*Mtb* infection for histopathological analysis.

### 2.7. Statistical Analysis

Data were analyzed using FlowJo software (version 10.7.1) (flow cytometry), HLA Fusion (version 4.2.0) (HLA typing gels) or GraphPad Prism 6 (version 6.01) software. Data were considered significant if the *p*-values obtained using a two-way ANOVA test or unpaired multiple *t*-test were < 0.05. Significant differences are noted as * *p* < 0.05, ** *p* < 0.01, *** *p* < 0.001, **** *p* < 0.0001 or n.s. (not significant). Data were expressed as mean ± SEM.

## 3. Results

### 3.1. huDRAG-A2 Mice Show Significantly Improved Human Immune Cell Reconstitution in the Blood Compared to huNRGs

**Figure 1:**
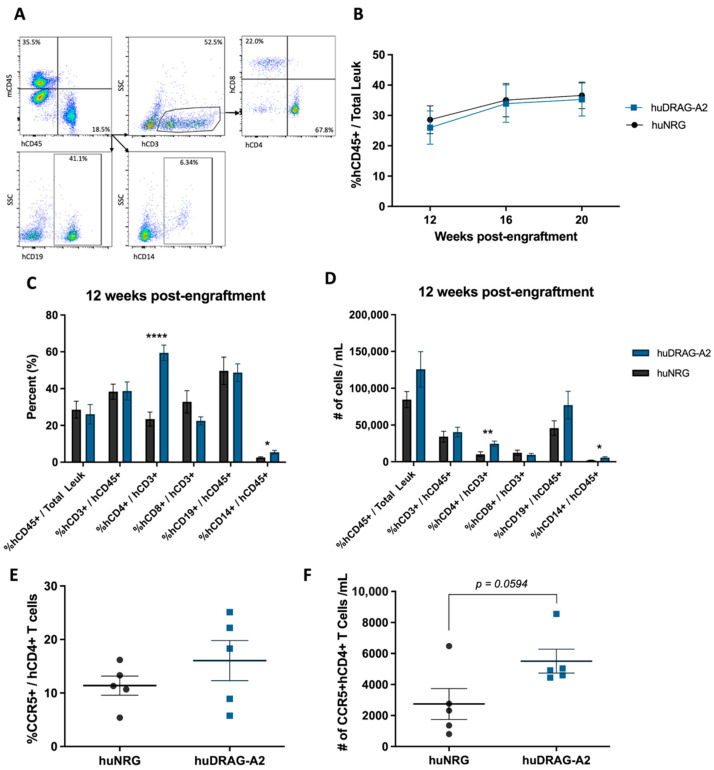
*Humanized DRAG-A2 mice show significantly improved human CD4+ T cell and CD14+ monocyte reconstitution compared to huNRG mice.* (**A**) Gating strategy for the quantification of human immune cell populations shown on an example of a successfully engrafted huDRAG-A2 mouse; (**B**) mean absolute hCD45+ leukocyte count per mL of whole blood of all huNRG (N = 18) and huDRAG-A2 (12 weeks N = 23, 16–20 weeks N = 22) mice at 12-, 16- and 20 weeks post-engraftment; (**C**) mean reconstitution (%) and (**D**) absolute number of human immune cells in the blood of huNRG (N = 18) and huDRAG-A2 (N = 23) mice at 12 weeks post-engraftment; (E) mean expression (%) and (F) absolute number of CCR5+ human CD4+ T cells in the blood of huNRG and huDRAG-A2 (N = 5 each) at 20 weeks post-engraftment. Data are expressed as mean +/− SEM, *p* value = * <0.05, ** <0.01, **** <0.0001.

Human leukocyte engraftment, as well as human immune cell subsets, were measured in the blood of hu-mice using flow cytometry. First, cells were stained with mouse and human CD45 to identify the human leukocyte population, followed by CD3+, CD4+ and CD8+ T cells, CD19+ B cells and CD14+ monocytes (Figure 1A). Both humanized NRG and DRAG-A2 mice developed robust human leukocyte reconstitution between 20% to 35% human CD45+ leukocytes out of total leukocytes in the blood by 12 weeks post-engraftment following reconstitution with human CD34+ HSCs (Figure 1B). The human leukocyte population was further analyzed to measure the frequency of human immune cell subsets in the blood. Both the mean frequency and absolute number of human CD4+ T cells and CD14+ monocytes were significantly higher in huDRAG-A2 mice compared to huNRGs (Figure 1C,D). Both models had more than 10% of human CD4+ T cells expressing the HIV-associated co-receptor CCR5, and there was a clear trend towards a higher absolute number of human CD4+ T cells expressing CCR5 in the blood of huDRAG-A2 mice compared to huNRG mice at 20 weeks post-engraftment (Figure 1E,F).

### 3.2. huDRAG-A2 and huNRG Mice Infected with HIV Demonstrate Hallmark Characteristics of Infection

**Figure 2:**
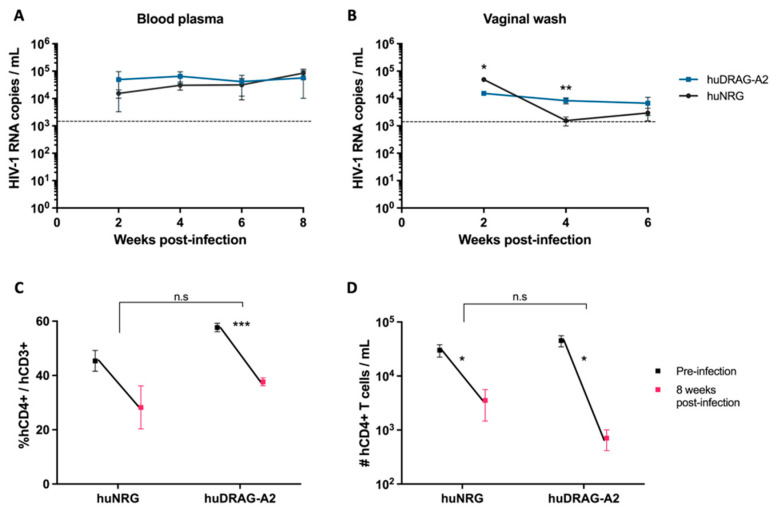
Both huNRG and huDRAG-A2 mice can sustain HIV infection, and both display a drop in CD4+ T cells 8 weeks post infection. Viral load in the (**A**) blood plasma and (**B**) vaginal wash of huNRG (N = 5) and huDRAG-A2 (N = 3) mice at 2, 4, 6 and 8 weeks post-intravaginal HIV infection; (**C**) percent and (**D**) absolute human CD4+ T cell frequency pre- and 8 weeks post-infection in the blood of huNRG (N = 5) and huDRAG-A2 (N = 3). No significant differences were observed in CD4+ T cell depletion between the huNRG and huDRAG-A2 groups. Data for huNRG and huDRAG-A2 are combined from 2 separate experiments and are expressed as mean +/− SEM, n.s = not significant, *p* value = * <0.05, ** <0.01, *** <0.001. Dotted line indicates the threshold of detection of 1500 HIV-1 RNA copies/mL.

**Figure 3:**
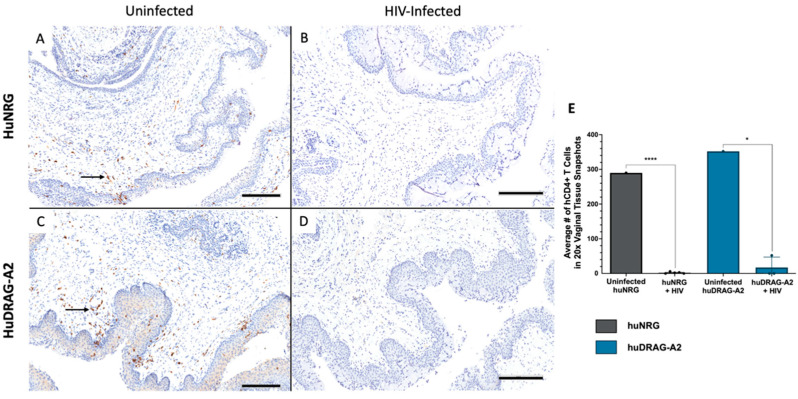
*Intravaginal HIV infection results in depletion of CD4+ T cells in the vaginal mucosa of huNRG and huDRAG-A2 mice.* (**A**) Representative uninfected huNRG vaginal mucosa, and (**B**) HIV-infected vaginal mucosa at 8 weeks post-infection stained for human CD4+ by IHC (observed in N = 5) (**C**) Representative uninfected huDRAG-A2 vaginal mucosa, and (**D**) HIV-infected huDRAG-A2 at 8 weeks post-infection stained for human CD4+ T cells by IHC (observed in N = 3). Black arrows indicate hCD4+ T cells stained brown. (**E**) Quantification of human CD4+ T cells in the vaginal mucosa of uninfected huNRG and huDRAG-A2 mice (N = 1 each), and HIV-infected huNRG (N = 5) and huDRAG-A2 (N = 3) mice. Data are expressed as mean +/− SEM, *p* value = * <0.05, **** <0.0001. (All images at 10×, scale bar = 200 μm).

**Figure 4:**
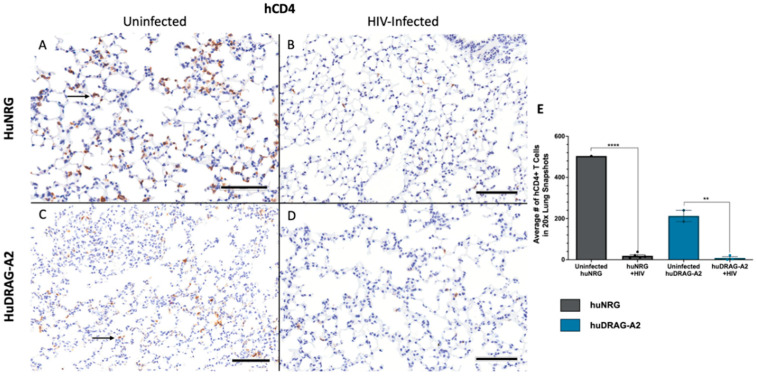
Human CD4+ T cells are depleted in the lung of huNRG and huDRAG-A2 mice at 8 weeks post-infection, indicating viral dissemination. (**A**) Representative uninfected and (**B**) HIV-infected huNRG lung stained for human CD4+ by IHC at 8 weeks post-infection (observed in N = 5). (**C**) Representative uninfected and (**D**) HIV-infected huDRAG-A2 lung stained for human CD4+ by IHC at 8 weeks post-infection (observed in N = 3). (**E**) Quantification of human CD4+ T cell depletion in the lung tissue of uninfected huNRG (N = 1) and huDRAG-A2 mice (N = 2), and HIV-infected huNRG (N = 5) and huDRAG-A2 (N = 3) mice. Black arrows indicate hCD4+ T cells stained brown. Data are expressed as mean +/− SEM, *p* value = ** <0.01, **** <0.0001. (All images at 20×, scale bar = 100 μm).

**Figure 5:**
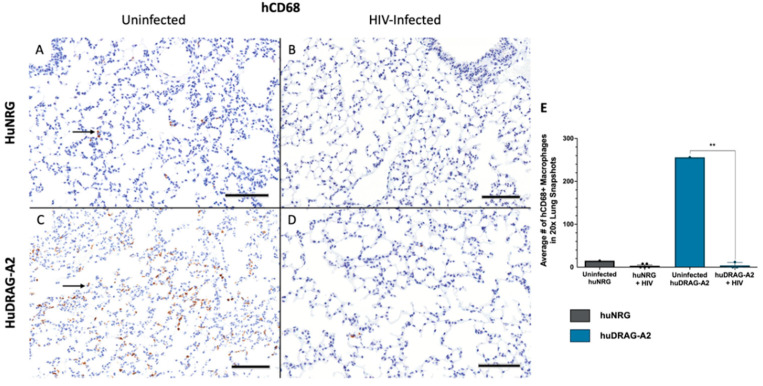
Human CD68+ macrophages are depleted in the lung of huNRG and huDRAG-A2 mice at 8 weeks post-infection, indicating viral dissemination. (**A**) Representative uninfected and (**B**) HIV-infected huNRG lung stained for human CD68+ by IHC at 8 weeks post-infection (observed in N = 5). (**C**) Representative uninfected and (**D**) HIV-infected huDRAG-A2 lung stained for human CD68+ by IHC at 8 weeks post-infection (observed in N = 3). (**E**) Quantification of human CD68+ in the lung tissue of uninfected huNRG and huDRAG-A2 mice (N = 1 each), and HIV-infected huNRG (N = 5) and huDRAG-A2 (N = 3) mice. Black arrows indicate hCD68+ macrophages stained brown. Data are expressed as mean +/− SEM, *p* value = ** <0.01. (All images at 20×, scale bar = 100 μm).

Well-engrafted (%hCD45 of total leukocytes > 10%) female huNRG and huDRAG-A2 mice were infected intravaginally with HIV-1 to confirm susceptibility to infection. huNRG mice with similar reconstitution levels to the huDRAG-A2s were selected in order to prevent variation in outcomes due to differences in the number of target cells (Table S3). At 2 weeks post-infection, both the huNRG and huDRAG-A2 mice showed detectable viral load in the blood plasma (Figure 2A). Despite higher viral load in the vaginal wash of the huNRGs at the earlier time point of 2 weeks post-infection, by 4 weeks post-infection, the viral load was significantly higher in the huDRAG-A2s (Figure 2B). Viral load was first detected in the plasma starting at 2 weeks post-infection and was maintained over the subsequent weeks, indicating viral dissemination into the bloodstream and the peripheral tissues (Figure 2A,B). There was a significant decrease in frequency and number of human CD4+ T cells in the blood of both the huNRG and huDRAG-A2 mice at 8 weeks post-infection, which is a hallmark of HIV infection in humans (Figure 2C,D). There was no statistically significant difference between loss of CD4+ T cells in huNRG and huDRAG-A2 mice. This observation was also confirmed in the tissues using immunohistochemistry as it is well-known that the depletion of T cells in mucosal sites is observed in early HIV pathogenesis. Prior to infection, both the huNRG and huDRAG-A2 mice showed a similar abundance of human CD4+ T cell infiltration within the vaginal mucosa of both huNRG and huDRAG-A2 mice (Figure 3A,C). Additionally, both human CD4+ T cells and CD68+ macrophage were substantially repopulated within the lung (Figure 4A,C and Figure 5A,C) and spleen (Figure S2A,C). When compared to the uninfected controls, the frequency of human CD4+ T cells in the vaginal mucosa, spleen and lung, and CD68+ macrophages in lung tissue of both the huNRG and huDRAG-A2 mice was significantly reduced at 8 weeks post-infection (Figure 3, Figure 4 and Figure 5 and Figure S2). In particular, almost no CD4+ T cells were present in the vaginal tissue 8 weeks post-infection (Figure 3).

### 3.3. HuDRAG-A2 Mice May Better Recapitulate Granuloma Structure and Immune Cell Involvement Characteristic of Human TB Compared to huNRGs

**Figure 6:**
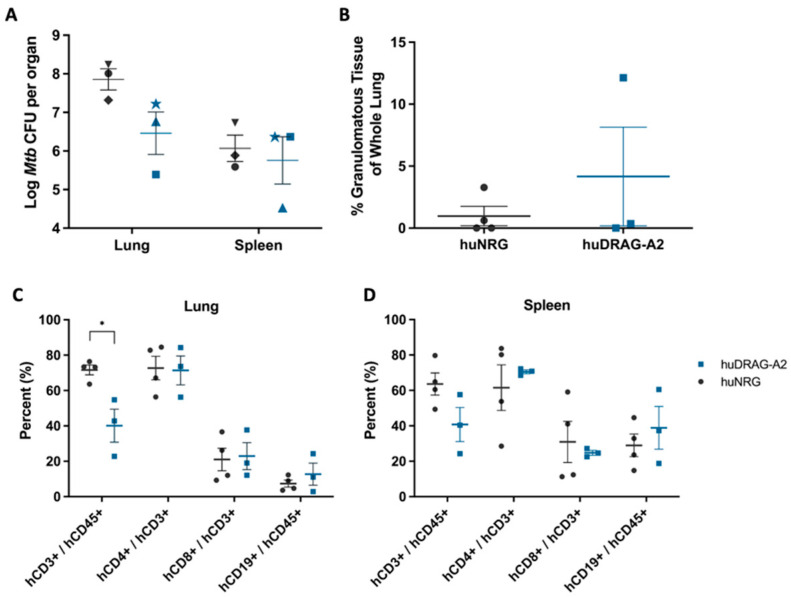
Following H_37_Rv *Mtb* infection, both huNRG and huDRAG-A2 mice show high bacterial load in the lung, as well as dissemination to the spleen with human immune cell subsets present within these tissues. (**A**) *Mtb* bacterial load in the lung and spleen of huNRG and huDRAG-A2 mice (N = 3 each), symbols indicate individual mice; (**B**) the percentage of granulomatous tissue in the whole lung of huNRG (N = 4) and huDRAG-A2 (N = 3) mice; (**C**) the frequency of humanT and B cells in the lung and, (**D**) spleen of huNRG (N = 4) and huDRAG-A2 (N = 3) mice at 4 weeks post-infection with H_37_Rv *Mtb*. Data for huNRG are combined from 3 separate experiments and are expressed as mean +/− SEM, *p* value = * <0.05.

Since HIV/*Mtb* co-infection is a major health concern worldwide, it is important that animal models of HIV can also recapitulate human *Mtb* infection and pathology. Therefore, it is of interest to compare the current and next generation huNRG and huDRAG-A2 in the context of TB. Both huNRG and huDRAG-A2 mice were infected with the H_37_Rv strain of *Mtb* and developed similar bacterial loads in the lungs as well as dissemination to the spleen at 4 weeks post-infection (Figure 6A). Although there was variability between individual mice, both huNRG and huDRAG-A2 mice formed granulomatous tissues within the lungs (Figure 6B). Flow cytometric analysis of the lung and spleen of huNRG and huDRAG-A2 mice at the experimental endpoint showed that despite the overall human CD3+ T cell frequency in the blood of the huDRAG-A2 mice being higher than the huNRGs pre-infection (Table S4), the CD3+ T cell population is significantly lower in the lung of the huDRAG-A2s following *Mtb* infection compared to the huNRGs (Figure 6C,D). These data suggest T cell levels were reduced in the lung during *Mtb* infection. Although the mechanism is not clear, *Mtb* is thought to cause apoptosis of certain subsets of T cells in the blood and lungs of humans with TB [40,41,42,43]. Preliminary data also indicate certain macrophage phenotypes (CD11b+, CD206+, CD14+, CD14-CD169+ alveolar macrophages and CD14+CD169- interstitial macrophages) are present within the lungs of both huNRG and huDRAG-A2 mice infected with TB (Figure S3).

**Figure 7:**
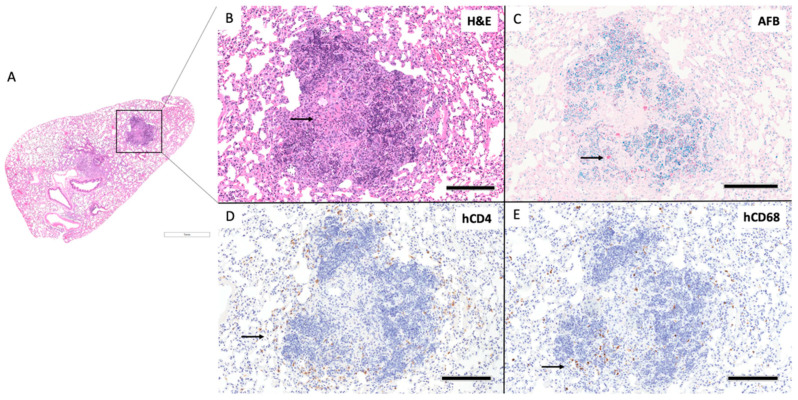
HuDRAG-A2 mice show classically organized granuloma formation with human immune cell involvement at 4 weeks post-infected with H_37_Rv *Mtb*. (**A**) Whole lung section H&E (2×). (**B**) granuloma H&E (10×), arrow indicates foci of central caseating necrosis, (**C**) granuloma AFB (10×), arrows indicate *Mtb* bacilli stained red, (**D**) human CD4+ by IHC (10×), arrow indicates human CD4+ T cells stained brown, (**E**) human CD68+ by IHC (10×), arrow indicates human CD68+ macrophages stained brown; (scale bars for 2× = 1 mm; 10× = 200 µm).

**Figure 8:**
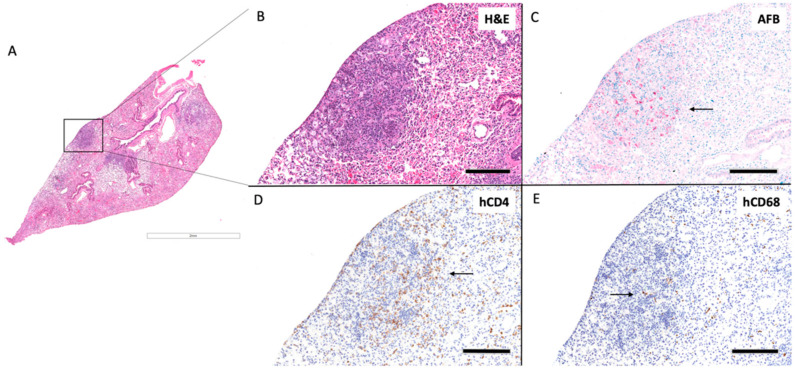
HuNRG mice develop granulomas with human immune cell involvement at 4 weeks post-infected with H_37_Rv *Mtb*. (**A**) Whole lung section H&E (2×). (**B**) Granuloma H&E (10×), (**C**) granuloma AFB (10×), arrow indicate *Mtb* bacilli stained red, (**D**) human CD4+ by IHC (10×), arrow indicates human CD4+ T cells stained brown, (**E**) human CD68+ by IHC (10×), arrow indicates human CD68+ macrophages stained brown; (scale bars for 2× = 1 mm; 10× = 200 µm).

Out of three huDRAG-A2 mice infected with H37Rv *Mtb*, one developed well-formed classical granulomas, while another developed small ill-formed granulomas at 4 weeks post-infection, and the last granulomas were not present, but focal mild inflammatory infiltrate were observed. The well-organized granuloma in the huDRAG-A2 lung also showed focal caseating necrosis within the center of the granuloma with severe karyorrhexis surrounding the caseum (Figure 7B). AFB stains showed *Mtb* bacilli being contained within the granuloma structure with hCD68+ macrophages scattered throughout and hCD4+ T cells surrounding the granuloma in a halo structure (Figure 7C–E). One out of four *Mtb*-infected huNRG mice developed well-organized granulomas (Figure 8B) with another that developed small ill-formed granulomas at 4 weeks post-infection. AFB staining showed *Mtb* localized within the granuloma structure while hCD4 and hCD68 IHC staining showed hCD4+ T cells and hCD68+ macrophages scattered throughout the granuloma structure (Figure 8C–E).

**Figure 9:**
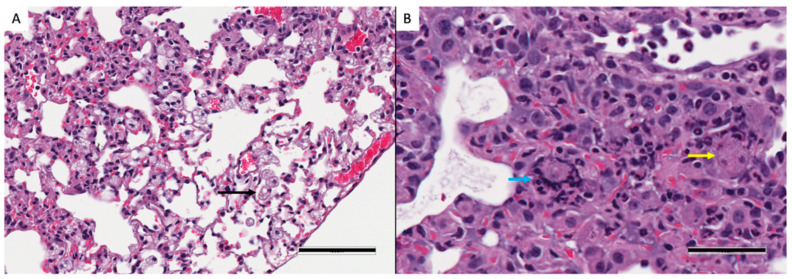
Features of granuloma pathology within huDRAG-A2 mouse at 4 weeks post-Mtb infection. (**A**) H&E stains of lung pathology illustrating foamy alveolar macrophages within airways indicated by a black arrow (20×, error bar represents 100 µm; observed in huNRG N = 4, and huDRAG-A2 N = 3). (**B**) H&E stains showing pathologically differentiated cells such as a multinucleated giant cell and a foamy macrophage within a granuloma (indicated by a blue and yellow arrow, respectively; 40× error bar represents 50 µm; observed in lung granulomas of huNRG N = 1, and huDRAG-A2 N = 1).

In both *Mtb*-infected huNRG and huDRAG-A2 mice, AFB staining was most prominent in granuloma structures with very few bacilli throughout the rest of the lung, similar to what is seen in *Mtb*-infected human tissues (Figure 7C and Figure 8C). HuNRG and huDRAG-A2 mice that did not show granulomas in histology sections still had other pathological lung features of TB disease such as chronically inflamed tissue and inflammatory infiltrates of lymphocytes. All *Mtb*-infected mice developed foamy macrophages within the airways; in previous literature, these cells were classified in humanized mice as foamy alveolar macrophages (Figure 9A) [44]. In the huNRG and huDRAG-A2 mice that developed well-organized granulomas, many foamy macrophages, along with some multinucleated giant cells, were found within the granuloma structures (Figure 9B).

### 3.4. HIV/Mtb Co-Infection Shows Trend of Worsened Pathology in Co-Infection

**Figure 10:**
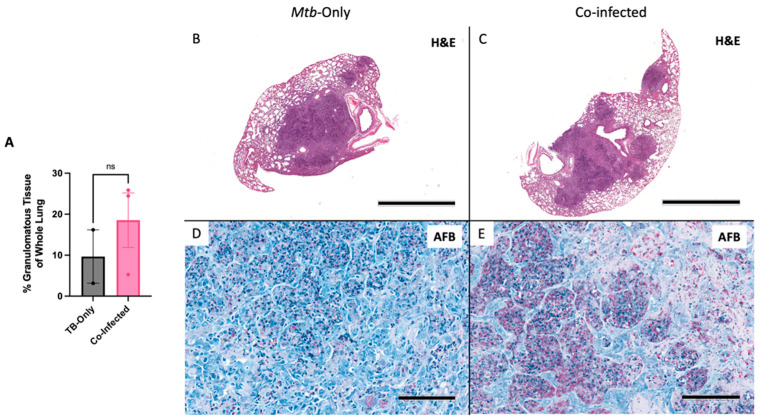
HuNRG mice are capable of sustaining HIV/*Mtb* co-infection and develop severe granulomatous lesions with abundant *Mtb* bacilli. (**A**) Percent of granulomatous tissue in the whole lungs of *Mtb* only infected and HIV/*Mtb* co-infected huNRG mice. Whole lung section of the highest percentage of lung granulomatous lesions found in (**B**) the *Mtb*-single infection group, and (**C**) HIV/*Mtb* co-infected group (scale bars represent 2 mm). Acid fast bacilli stains showing *Mtb* bacilli populated within granulomas of (**D**) *Mtb*-only infected and (**E**) co-infected huNRG lung granulomas (observed in *Mtb*-only N = 2, and HIV/*Mtb* co-infected N = 3; AFB images are at 20×, scale bars = 100 µm). Data are expressed as mean +/− SEM, ns = not significant.

An HIV/*Mtb* co-infection pilot study was conducted with huNRG mice to investigate the potential of HSC-method generated humanized mice to be used for co-infection studies. Upon confirming successful HIV-1 infection in the plasma of the co-infected huNRG group (Figure S4), all animals in the study were infected with low-dose *Mtb* intranasally at 3.5 weeks post-HIV infection. At the experimental endpoint, (4.5 weeks post *Mtb*-infection, or 8 weeks post-HIV infection), all huNRG mice in both the *Mtb*-only and co-infected groups developed multiple granulomatous lesions (Figure 10A–C). Although not significant, the HIV/*Mtb* co-infected group showed more granulomatous lesion formation in the lungs (Figure 10A). All the granulomatous legions in both groups were densely populated with *Mtb* bacilli within the granuloma structures (Figure 10D,E).

## 4. Discussion

This study compared the current generation HSC-method huNRG and a next-generation HLA transgenic model in terms of their immune reconstitution, disease course and tissue responses/histology for HIV and *Mtb* infection. This step is important to assess the utility of these models for use in HIV, TB or HIV/TB co-infection studies. Developing models that better recapitulate response to human pathogens can enable us to ask complex immune questions and develop novel therapeutics and vaccines. We demonstrated that current-generation humanized mice (huNRG) and next-generation transgenic humanized mice (huDRAG-A2) both repopulate with human immune cells and can be successfully infected by and sustain both HIV and *Mtb* infection. Although huNRG mice have been used for a range of infectious disease studies, including HIV and TB, newer generations of hu-mice have demonstrated the potential to better recapitulate the human immune system. In particular, HLA-transgenic hu-mice can help address issues with HLA/mouse MHC mismatch upon human stem cell engraftment. Varieties of background models with different HLA transgenes such as HLA-A2, HLA-DRB1*15, HLA-DRB1*04:05, and HLA B*51:01 have all demonstrated improvements in human immune development over their non-transgenic counterparts upon engraftment with HLA-matched HSCs or fetal cells [17,18,19,20]. In more direct relevance to this study, the transgenic NRG strain, termed DRAG mice (expressing the HLA-DRB1*04:01 transgene), have shown improvements over the NRG strain using the HSC-only method of engraftment [15]. A previous comparison between huDRAG and huNRG mice showed significant improvements in the repopulation of the lymphoid lineage with enhanced functionality producing higher levels of cytokine and isotype class-switched antibodies [15]. The huDRAGA model, which expresses an additional HLA class I transgene (HLA-A*02:01), reported advantages comparable to the huDRAG over the huNRG, while also demonstrating improved cytotoxic CD8+ T cell function [16]. These HLA-transgenic models illustrate the significance of the role of HLA-matching in recapitulating human immune responses with humanized mice in vivo.

In this study, we have crossed DRAG mice to NRG-A2 mice to create DRAG-A2 mice, and subsequently engrafted the mice with HLA-matched HSCs. The results from the direct comparison between the huDRAG-A2 and huNRG in this study demonstrate that although both huNRG and huDRAG-A2 mice reconstitute with significant immune cell populations, huDRAG-A2 mice may offer additional advantages. HuDRAG-A2 mice reconstituted with significantly higher counts of human CD4+ T cells and CD14+ monocytes (Figure 1C,D). This finding is of particular interest for pathogens such as HIV and *Mtb*, as not only are CD4+ T cells and macrophages target cells of HIV in mucosal tissues, they also play major roles in the response to infection, disease progression, and pathology in TB [45,46,47]. Further investigation of CD4+ T cell and CD68+ macrophage reconstitution within lung tissue was of interest as it is the site of initial infection for *Mtb* as well as co-localization during co-infection for HIV [33,45]. Immunohistochemistry staining of huNRG and huDRAG-A2 lung sections revealed that both models display clear infiltration of CD4+ T cells and CD68+ macrophages into lung tissue. Additionally, both these models express the HIV-associated co-receptor CCR5 on a significant portion of their CD4+ T cells, with the huDRAG-A2 appearing to trend towards a higher absolute number of CCR5+ CD4+ T cells in the blood at 20 weeks post-engraftment (Figure 1E,F). These results validate both the huNRG and huDRAG-A2 as good models for HIV and TB studies and further highlights that the huDRAG-A2 mice have the potential to better recapitulate human immune responses as a pre-clinical in vivo model for certain infectious disease investigations.

HIV is primarily a sexually transmitted disease, and as such, target cells in the vaginal or rectal mucosa are essential for productive infection. Both models of hu-mice we described are susceptible to intravaginal infection, as indicated by the presence of HIV RNA in the blood plasma starting at 2 weeks post-intravaginal HIV infection (Figure 2A). The viral load detected in the blood plasma of our mice was comparable to similar hu-mouse models of HIV published in previous literature [21,37,48,49,50,51,52]. By measuring viral RNA in the vaginal wash and blood plasma at 2, 4, 6 and 8 weeks post-infection, we were able to track viral dissemination from the initial site of infection, the vaginal mucosa, into the bloodstream, which indicates successful HIV infection (Figure 2A,B). High viral loads are expected in the vaginal lavage shortly following intravaginal infection, but by 4 weeks post-infection, the viral load was reduced in the vaginal wash. At the same time point and later, the viral load in the blood plasma plateaued and was maintained until the conclusion of the experiment at 8 weeks post-infection. This recapitulates infection in humans, where the viral load increases rapidly before stabilizing to a viral set point, and the viral load is often maintained relatively consistently for several years before the infection progresses to AIDS [53]. This is thought to occur within the first 4 to 24 months following infection and can be a useful predictor of disease progression [53]. In humanized mice, the viral set point has not been well characterized, but we appear to reach a level that is quite consistent and steady in the 8 weeks post-HIV infection. In other hu-mouse models, this time point of 8 weeks has been suggested to mimic early chronic infection in humans [54].

HuDRAG-A2 and huNRG mice infected with HIV also showed significant depletion in human CD4+ T cells in the blood at 8 weeks post-infection (Figure 2C,D). Furthermore, a similar reduction in CD4+ T cells in both the vaginal mucosa and lung, as well as a reduction in CD68+ macrophages in the lung tissue of these mice was observed compared to uninfected controls using immunohistochemistry (Figure 3 and Figure 4). This depletion was quite severe in the tissues with no visible cells in the field of view (20×). CD4+ T cell depletion is a hallmark characteristic of uncontrolled HIV infection in humans, but depletion of macrophages also contributes to disease progression [46]. As the initial site of infection, human CD4+ T cells in the vaginal mucosa were likely targeted and depleted in the early stages of HIV infection. Although not the primary site of infection, the lungs are known to be an important site of the early stages of acute HIV infection (Corleis et al., 2019, Santangelo et al., 2015, Kalsdorf et al., 2013), and would be of relevance for HIV/*Mtb* co-infection. Non-human primate models of Simian Immunodeficieny Virus (SIV) infection have indicated that SIV replication in BAL cells of rhesus macaques is detectable as early as 10 days following systemic infection (Barber et al., 2006). Clinical studies have also detected HIV in monocytes, bronchoalveolar leukocytes and alveolar macrophages collected from the BAL of both asymptomatic and AIDS-stage people living with HIV [46,55,56,57,58,59,60].

Although these antigen-specific immune responses were not within the scope of these experiments, previous literature characterizing the huDRAG mice suggests that our huDRAG-A2 model may support a more well-rounded human immune system suitable for vaccine testing and development [15,16,61]. CD4+ helper T cell function is thought to be essential for generating antibody responses when exposed to a pathogen, and as such, enhanced CD4+ T cell function indirectly results in improved B cell function. CD4+ T cells were found to be reconstituted at significantly higher rates in the huDRAG-A2 model compared to the huNRGs (Figure 1C,D), and similar HLA-transgenic models have shown to have improved cytokine and antibody production [15,16,62,63]. However, this needs to be investigated further in future studies.

Unlike HIV, TB research can be performed in standard mouse models since it infects mouse cells, and as such, hu-mouse models have been less commonly used for studies solely investigating *Mtb* [30,32]. Current generation hu-mice (huNRG-HSC, huNSG-BLT) have been successfully infected by the H_37_Rv strain of *Mtb* and mount immune responses to infection [30,32]. Here, we found that upon intranasal infection with the H_37_Rv strain, both the huNRG and huDRAG-A2 mice demonstrated a high bacterial burden in the lung and dissemination to the spleen 4 weeks post-infection, which agrees with previous findings using similar HSC or BLT models (Figure 6A) [29,30]. Disease course was similar in both models in terms of effects on the health of the mouse. There were no significant differences in weight loss between the groups, and only one huNRG mice reached the pre-defined humane 20% weight loss endpoint by 4 weeks post-infection (Figure S5).

Immunohistochemistry of the lung granulomas of huNRG and huDRAG-A2 mice following intranasal infection with the H_37_Rv strain of *Mtb* revealed that both human CD4+ T cells and CD68+ macrophages were involved in the formation of granulomas and the containment of the mycobacterium (Figure 7D,E and Figure 8D,E). In the huDRAG-A2 mice, the granulomas were more organized in the classical structure, where macrophages were scattered throughout the granuloma and surrounded small foci of caseating necrosis, while the entire granuloma structure was surrounded by a hCD4+ T lymphocyte halo (Figure 7) [64]. AFB staining used to visualize *Mtb* bacilli revealed localization of *Mtb* mostly within the granuloma lesions with scarce bacilli throughout the rest of the lung tissue (Figure 7C and Figure 8C). Despite failing to observe granulomatous lesions in the lungs in some of the *Mtb*-infected huNRG and huDRAG-A2 mice, they were confirmed to be infected by high levels of lung CFU (Figure 6A) and thus indicate that histological granuloma structures may have been missed during histological sectioning. Lung pathology further illustrated the development of foamy alveolar macrophages in airways (Figure 9) [44]. Although the huDRAG-A2 model may have developed better granuloma organization with a clearer focal necrosis and a small central caseum, both the huNRG and huDRAG-A2 models have demonstrated the capability of developing granulomas and lung pathology that are representative of major features of human TB disease. It is also notable that while human-like granuloma formation was not consistently seen in all mice, this variability and heterogeneity are also observed in humans infected with TB that develop various stages of granulomas ranging from well-organized to poorly formed [65,66]. In addition, in the *Mtb*-only infected humanized mice, true caseating granulomas or fibrous cuffs were rarely seen, which are features that are more frequently observed in human granulomas.

Further analysis into immune cell subsets within the lung and spleen of both huNRG and huDRAG-A2 mice infected with H_37_Rv using flow cytometry yielded results that may offer further insight into the dynamic milieu of the granuloma in vivo. Depletion of T cells is particularly important in the context of HIV/*Mtb* co-infection [67]. Despite starting with significantly higher T cell reconstitution in the blood compared to the huNRGs, human CD3+ T cells in the lung were significantly lower in the huDRAG-A2 mice compared to the huNRGs (Figure 6B). Although it is unclear the cause of this observation, this may imply that the decrease in T cell frequency was due to the *Mtb* infection taking a greater effect in inducing apoptosis of human CD3+ T cells within the huDRAG-A2, and that since this is observed in the blood and lungs of humans, this model may better recapitulate the human pathology presentation [40,41]. 

To our knowledge, this is the first report of the use of HLA-transgenic humanized models, such as the huDRAG-A2 model, for *Mtb* infection. Both the huNRG and huDRAG-A2 are good models for recapitulating HIV and TB infection in vivo. This serves as the foundational stepping stone for future immune functionality and mechanism studies within the context of HIV and/or *Mtb* infection.

To explore the feasibility and utility of the HSC-method hu-mouse model in investigating HIV/*Mtb* co-infection, female huNRG mice were used for a proof-of-concept pilot experiment with a co-infection group and *Mtb*-only single infection control. Co-infection of huNRG mice was modeled from our single infection studies and methods with primary intravaginal HIV-1 infection followed by subsequent intranasal *Mtb* infection. At 4.5 weeks post-*Mtb* co-infection, huNRG mice in both the co-infected group and single-*Mtb* infected group developed substantial granulomatous lesions (Figure 10). Due to prior literature, it was expected that TB disease pathology would be significantly exacerbated with primary HIV-1 infection [33], and a trend of more granulomatous lesions in the co-infected group was observed, which missed significance likely due to low Innumbers. Lack of significance may also have been impacted by another factor. Upon literature review, the depot-medroxyprogesterone acetate (DMPA) that was used to induce diestrus for intravaginal HIV infection may also exacerbate TB disease progression. Previous literature has shown the detrimental effects of DMPA on *Mtb* containment by down-regulating cytotoxic lymphocyte activities [68,69]. As a result, the *Mtb*-only control group may also have experienced worsened disease due to receiving DMPA. Additionally, given this exacerbated disease development caused by DMPA, the experimental endpoint of 4.5 weeks post-*Mtb* co-infection may have allowed both the singly-*Mtb* infected group and co-infected groups to both progress to severe pathology past where any differences in the rate of disease progression could be observed. Nevertheless, this pilot study shows the potential of an HSC-method model being used in future HIV/*Mtb* co-infection experiments. In future studies, we will infect with HIV systemically to avoid this complication.

*Study Limitations and Future Directions:* While these hu-mouse models were able to recapitulate many aspects of HIV and TB infection, every study has limitations. Both the huNRG and huDRAGA2 models do not have the full complement of immune cells in the same frequency that a human would have. For example, while T and B cell generation is substantial in our models, human myeloid cells such as macrophages and neutrophils are much lower than would be seen in humans due to a low level of cytokines such as GM-CSF/IL-3 that contribute to their differentiation. There are other models, such as the humanized MITRG/MISTRG, NSG-SGM3, and NOG-EXL, which have added cytokine transgenes to improve this population, although this can also lead to other drawbacks [13,70,71,72]. In the huDRAG-A2 mice we have utilized, we see enhancement of monocyte/macrophage levels in comparison to huNRG mice. Another limitation is the low sample size in some of our experiments (N = 3–5 for most HIV or TB experiments). These mice can be challenging to generate, especially when specific HLA-matched stem cells are required to reconstitute DRAG-A2 mice. In addition, the long timeframe for reconstitution (approximately 20 weeks for peak levels of engraftment) requires extensive time for development prior to use in studies. Lastly, these mice can also be susceptible to GvHD due to residual mature T cells from the stem cell preparations, which we have observed in approximately 10% of mice. A limitation in terms of disease pathology is that, unlike NHP, these mice do not develop latent TB and instead proceed to active TB fairly quickly after infection [73]. This is an area of interest in HIV/TB co-infection as due to the high prevalence of TB in endemic countries, many may have latent TB prior to becoming infected with HIV. Recently, a group created a substitute model of latency in humanized mice where mice were treated with the standard antibiotic regimen to model paucibacillary TB [44]. This will be an option to investigate in future studies where we could administer *Mtb* prior to HIV. Even though a relatively low dose of TB was used in the single-TB infection experiments (1 × 10^4^), we saw that some mice develop large granulomas or reach weight endpoints in a relatively short time frame of 4 weeks. This could limit the timeline for experimental endpoints post-infection, although ultra-low-dose *Mtb* infections should be explored to determine the effect on the endpoint. The next steps include repeating the HIV/TB co-infection study in both huNRG and huDRAG-A2 mice with systemic HIV-1 infection to avoid the use of DMPA as it could be a confounding factor in evaluating TB disease and pathology.

## 5. Conclusions

Our findings have demonstrated that both the huNRG and huDRAG-A2 models are able to sustain infection with either HIV or *Mtb* and, as such, are useful models for studying these pathogens. The huDRAG-A2 model may offer certain important advantages over the huNRGs for research questions that require higher numbers of immune cells and perhaps immune cell functionality. Additionally, both models display the ability to form necrosis within the granuloma following infection with *Mtb*. The huDRAG-A2 model, in particular, exhibited more classically well-organized granuloma structures observed in humans, such as the hCD4+ lymphocyte ring surrounding the granuloma. In a pilot study, huNRG mice were successfully co-infected with both HIV and *Mtb*, and preliminary data indicate that co-infected mice appear to have a trend toward worsened TB pathology. Since both the huNRG and huDRAG-A2 models offer a promising ability to recapitulate many aspects of both HIV and TB, it is important to further explore the models to become better informed in selecting the model that is best suited and most feasible for use in each experimental design. In future experiments, both these models will be co-infected with HIV and *Mtb* to investigate pathology, disease course and immune responses when compared to infection with either pathogen alone.

## Data Availability

Data is contained within the article or Supplementary Material.

## Supplementary Materials

The following supporting information can be downloaded at: www.mdpi.com/article/10.3390/v14091927/s1, Figure S1: Immunohistochemistry positive and negative controls; Figure S2: Human CD4+ T cells are depleted in the spleen of huNRG and huDRAG-A2 mice at 8 weeks post-infection, indicating viral dissemination; Figure S3: Human macrophage subsets in huNRG and huDRAG-A2 Lungs; Figure S4: HIV viral load in the blood plasma of HIV-infected mice prior to co-infection with *Mtb*; Figure S5: There was no significant difference in the survival of huNRG (N = 6) and huDRAG-A2 (N = 3) mice up to 4 weeks post-infection with H37Rv *Mtb*; Table S1: Flow cytometry antibodies used to characterize immune cell populations; Table S2: Cord blood samples used for humanizing NRG and DRAG-A2 mice; Table S3: Baseline pre-infection human immune cell reconstitution in the blood of humanized mice used for HIV infection. Table S4: Baseline pre-infection human immune cell reconstitution in the blood of humanized mice used for *Mtb* infection.
