# Supplementary material for: Comparing Current and Next-Generation Humanized Mouse Models for Advancing HIV and HIV/Mtb Co-Infection Studies
Source: Viruses. 2022 Aug 30;14(9):1927. doi: 10.3390/v14091927 (PMC9500899; doi:10.3390/v14091927)
Supplement: Supplementary file 1 [file viruses-14-01927-s001.zip › viruses-1881934-supplementary.pdf]

Supplementary Materials

METHODS:

Generation of NRG-A2 mice:

One male NSG-A2 mouse (stock ID# 009617, obtained from Jackson laboratories) was paired with a female NRG mouse (stock ID# 007799). From each offspring, a small piece of the tail was collected, DNA was extracted and *Rag* and HLA-A2 genotyping was done by PCR. After 12 generations of back-crossing with NRGs, the mice lacking the *Rag* allele and positive for HLA-A2 were maintained as a homozygous NRG-A2 colony.

Mouse Genotyping:

For *Rag* and *Scid* genotyping, Platinum Taq DNA Polymerase (Invitrogen) and the following primer sequences were used: *Rag* Forward primer: 5'-GAGGTTCCGCTACGACTCTG-3', *Scid* Forward primer: 5'-TGGATGTGGAATGTGTGCGAG-3', and a common primer (Forward primer: 5'-CCGGACAAGTTTTTCATCGT-3').

To confirm the presence of the HLA-A2 allele (A*02:01), PCR genotyping was performed using the MyTaq HS Red Mix (Bioline) and the following primer sequences: Forward primer: 5'-TTCTCCCTCTCCCAACCTATGTAG-3'; Reverse primer: 5'-CGACGACACTGATTGGCTTCT-3'.

To confirm the presence of the DRAG allele (DRB1*04:01), PCR genotyping was performed using the Platinum II Hot-Start PCR Master Mix (Invitrogen) and the following primer sequences were used: Forward primer: 5'-GTTTCTTGAGCAGGTTAAAC-3'; Reverse primer: 5'-CTGCACTGTGAAGCTCTCAC-3'; Forward internal control: 5'-CAAATGTTGCTTGTCTGGTG-3'; Reverse internal control: 5'-GTCAGTCGAGTGCACAGTTT-3'.

Flow cytometry antibodies:

Supplementary Table S1: Flow cytometry antibodies used to characterize immune cell populations.

**Marker**	**Clone**	**Colour**	**Concentration**	**Manufacturer**
Fixable viability dye	-	APC-eFluor 780	1 uL/mL	eBiosciences
mCD45	30-F11	Alexa Fluor 700	0.004 ug/mL	eBiosciences
hCD45	H130	Pacific Blue	0.01 ug/mL	eBiosciences
hCD3e	UCHT1	Qdot605	0.01 uM	eBiosciences
hCD4	RPA-T4	PerCP-Cy5.5	1.25 ug/mL	eBiosciences
hCD8a	RPA-T8	PE-Cy7	1.25 ug/mL	eBiosciences
hCD19	HIB19	PE	2.5 ug/mL	eBiosciences
hCD14	Tuk4	Alexa Fluor 647	0.01 ug/mL	eBiosciences
hCCR5	2D7	FITC	2 ug/mL	BD Biosciences
hCD11b	CBRM1/5	PerCP-Cy5.5	0.01 ug/mL	BioLegend
hCD14	M2E2	BV785	0.004 ug/mL	BioLegend
hCD206	7-239	APC	0.02 ug/mL	BioLegend
hCD169	15-2	PE	0.001 ug/mL	BioLegend
hCD16	3G8	FITC	0.01 ug/mL	BioLegend

Immunohistochemistry:

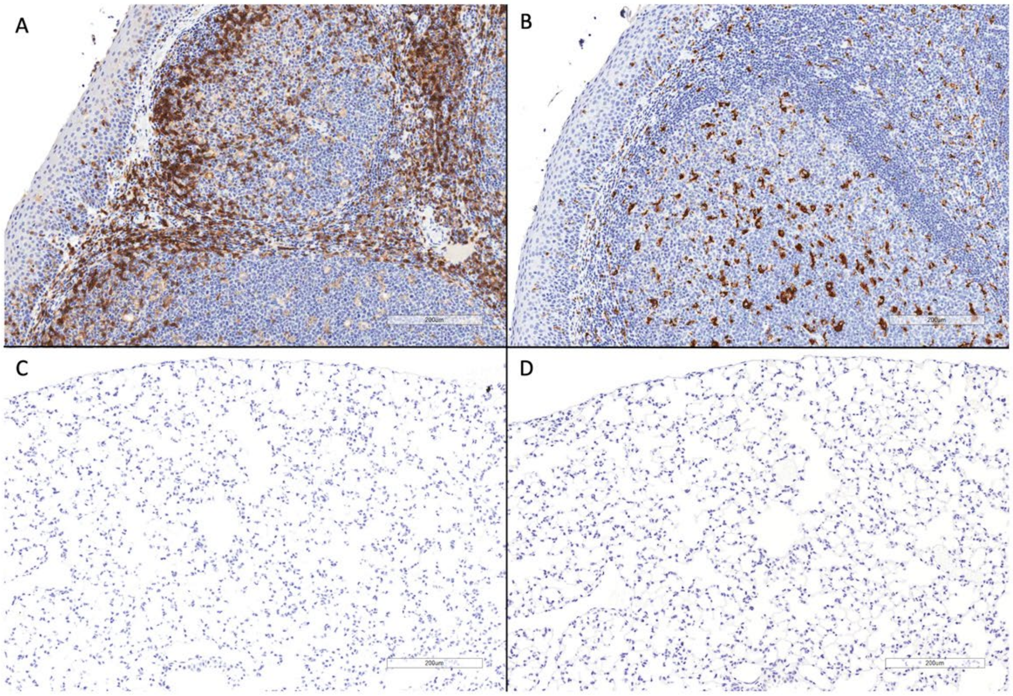

Supplementary Figure S1. *Immunohistochemistry positive and negative controls.* Top: Human tonsil **(A)** hCD4+ and **(B)** hCD68+ IHC positive controls. Bottom: Non-humanized NRG mouse lung **(C)** hCD4+ and **(D)** hCD68+ IHC negative controls (all 20x, scale bar = 200μm).

RESULTS:

Supplementary Table S2. Cord blood samples used for humanizing NRG and DRAG-A2 mice.

Cord blood sample #	HLA type	Mouse strain engrafted	Engraftment Method	Success rate
1	DR4+A2+	DRAG-A2	IH, newborn	6/6 (100%)
2	DR4-	NRG	IH, newborn	9/9 (100%)
3	DR4-	NRG	IH, newborn	9/9 (100%)
4	DR4-	NRG	IH, newborn	3/7 (42.8%)
5	DR4-	NRG	IH, newborn	6/7 (85.7%)
6	DR4+A2+	DRAG-A2	IV, adult	10/21 (47.6%)
7	DR4-	NRG	IH, newborn	11/12 (91.7%)
8	DR4+A2+	DRAG-A2	IH, newborn	6/9 (66.7%)
9	DR4+A2+	DRAG-A2	IH, newborn	9/11 (81.8%)

10	DR4-	NRG	IH, newborn	7/7 (100%)
11	DR4-	NRG	IH, newborn	9/9 (100%)
12	DR4-	NRG	IH, newborn	5/8 (62.5%)

Supplementary Table S3: Baseline pre-infection human immune cell reconstitution in the blood of humanized mice used for HIV infection.

Mouse #	Sample #	Strain	%hCD45+ of Total Leuk	#hCD45+/ mL	%hCD3+ / hCD45+	#hCD3+/ mL	%hCD4+ / hCD3+	#hCD4+/ mL
1	1	huDRAG-A2	79.56	449,300	26.5	119,065	55.1	65,605
2	1	huDRAG-A2	62.82	272,901	25.0	68,225	57.4	39,161
3	6	huDRAG-A2	21.3	75,280	68.1	51,240	60.5	31,000
4	2	huNRG	55.45	294,968	33.0	97,339	44.4	43,219
5	2	huNRG	71.11	237,307	38.7	91,838	55.0	50,511
6	7	huNRG	12.51	106,400	28.2	30,005	36.7	11,012
7	7	huNRG	19.22	81,200	72.5	58,870	53.3	31,378
8	7	huNRG	23.14	56,800	67.6	38.397	37.5	14,399

Supplementary Table S4: Baseline pre-infection human immune cell reconstitution in the blood of humanized mice used for *Mtb* infection.

Mouse #	Sample #	Strain	%hCD45+ of Total Leuk	#hCD45+ /mL	%hCD3+ / hCD45+	#hCD3+ /mL	%hCD4+ / hCD3+	#hCD4+ /mL	%hCD8+ / hCD3+	#hCD8+ /mL	%hCD14+ / hCD45+	#hCD14+ /mL
9	1	huDRAG -A2	65.65	87,224	68.4	59,662	77.7	46,357	16.8	10,023	2.06	1,797
10	1	huDRAG -A2	48.50	106,400	27.6	29,366	65.0	19,088	17.9	5,257	13	13,832
11	1	huDRAG -A2	34.23	110,800	52.4	58,059	68.7	39,887	22	12,773	6.34	7,025
12	3	huNRG	29.70	127,660	21.3	27,192	51.9	14,112	9.64	2,621	6.2	7,915
13	3	huNRG	19.97	196,940	20.0	39,388	51.9	20,442	8.49	3,344	3.58	7,050
14	10	huNRG	35.45	57,320	50.9	29,176	59.7	17,418	31.9	9,307	-	-

15	11	huNRG	6.39	38,280	96.8	37,055	76.8	28,458	20.3	7,522	-	-
16	12	huNRG	12.83	118,880	32.4	38,517	55.3	21,300	30.3	11,671	2.15	2,556
17	12	huNRG	22.73	108,400	66.4	71,978	59.3	42,683	35.9	25,840	2.83	3,068

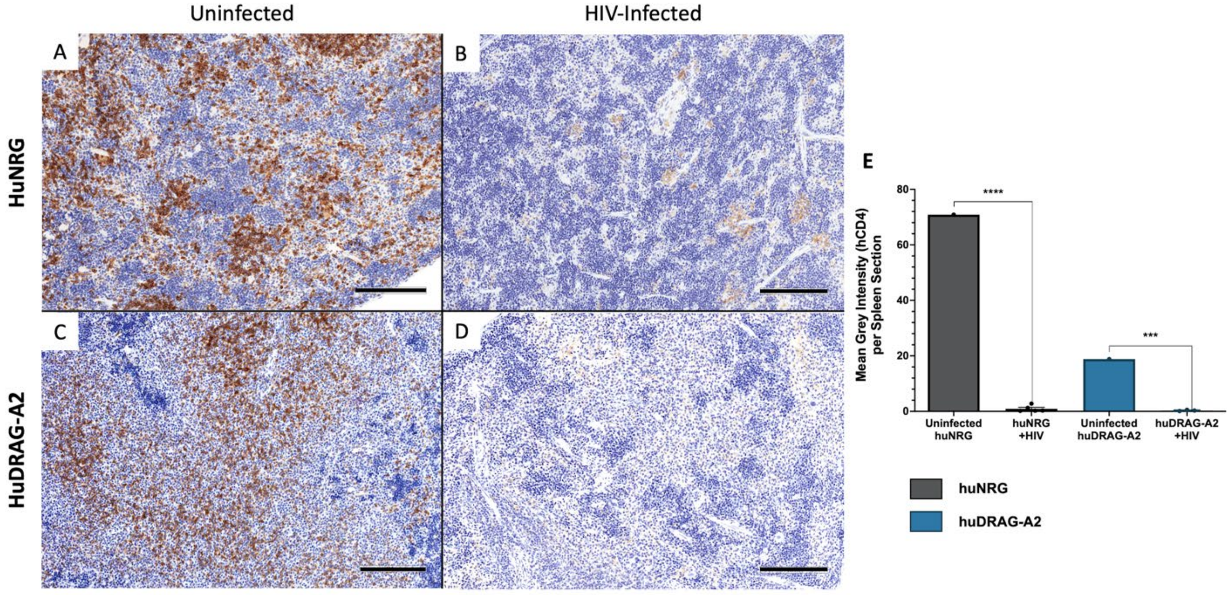

Supplementary Figure S2. *Human CD4+ T cells are depleted in the spleen of huNRG and huDRAG-A2 mice at 8 weeks post-infection, indicating viral dissemination.* (A) Uninfected and (B) HIV-infected huNRG lung stained for human CD4+ by IHC at 8 weeks post-infection (observed in N = 5). (C) Uninfected and (D) HIV-infected huDRAG-A2 lung stained for human CD4+ by IHC at 8 weeks post-infection (observed in N = 3). (E) Quantification of human CD4+ depletion in the spleen tissue of uninfected huNRG and huDRAG-A2 mice (N=1 each), and HIV-infected huNRG (N=5) and huDRAG-A2 (N=3) mice. Human CHuman CD4+ T cells are stained brown. D4+ T cells are stained brown. Data are expressed as mean +/- SEM, p value = *** <0.001, **** <0.0001. (All images at 10x, scale bar = 200 μm).

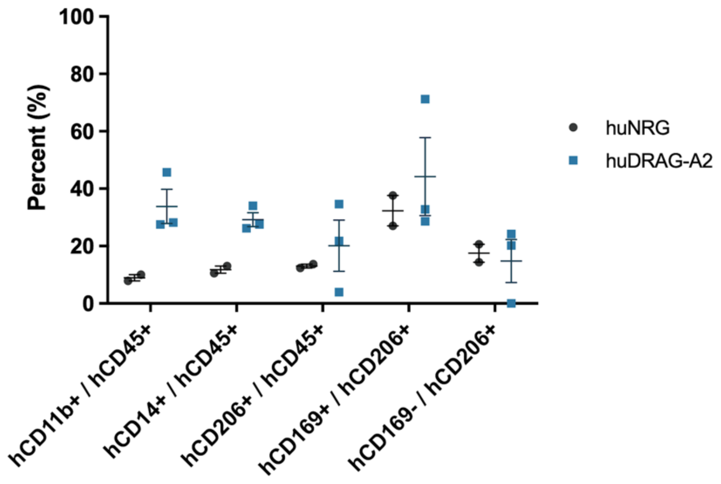

Supplementary Figure S3. *Human macrophage subsets in huNRG and huDRAG-A2 Lungs.* Various subsets of human macrophages were identified in the lungs of huNRG (N = 2) and huDRAG-A2 (N = 3) mice at 4 weeks post-TB infection. Data are expressed as mean +/- SEM.

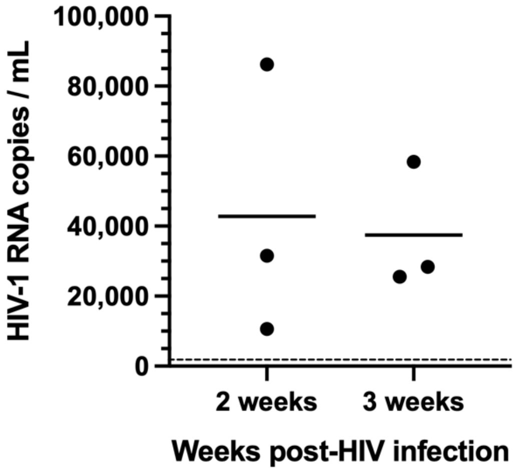

Supplementary Figure S4. *HIV viral load in the blood plasma of HIV-infected mice prior to co-infection with Mtb.* Successful HIV-1 infection was sustained in the blood plasma of huNRG mice at 2 and 3 weeks post-HIV infection (N = 3). Solid lines indicate means, dotted line indicates assay detection threshold at 1,500 RNA copies/mL.

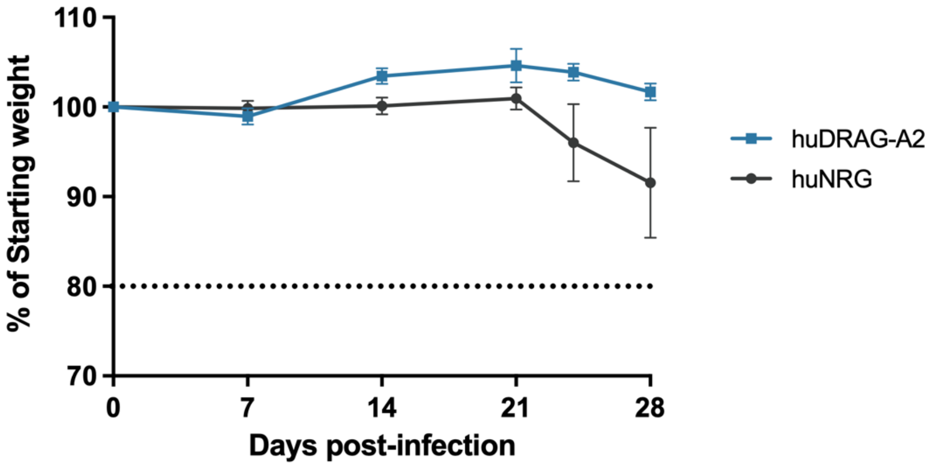

Supplementary Figure S5. *There was no significant difference in the survival of huNRG (N = 6) and huDRAG-A2 (N = 3) mice up to 4 weeks post-infection with H37Rv Mtb.* Dashed line indicates pre-determined humane 20% weight loss endpoint. Data are expressed as mean +/- SEM.
